# Narrative Group Intervention to Rediscover Life Wisdom Among Hong Kong Chinese Older Adults: A Single-Blind Randomized Waitlist-Controlled Trial

**DOI:** 10.1093/geroni/igab027

**Published:** 2021-08-02

**Authors:** Esther Oi Wah Chow, Sai-Fu Fung

**Affiliations:** Department of Social and Behavioural Sciences, City University of Hong Kong, Kowloon, Hong Kong

**Keywords:** Latent growth curve models, Narrative therapy, Randomized controlled trial, Tree of Life, Wisdom

## Abstract

**Background and Objectives:**

We developed a new group practice using strength- and meaning-based narrative therapy (NT) for older Chinese living in Hong Kong (HK), to enhance their life wisdom. This paper reports on the intervention and its short- and longer-term effectiveness.

**Research Design and Methods:**

A randomized waitlist-controlled trial was conducted. A total of 157 older adults were randomly recruited, of whom 75 were randomly assigned to the intervention group which received four 2-hr biweekly NT sessions using the “Tree of Life” metaphor. The others were placed on a waitlist. Perceived wisdom was assessed using the Brief Self-Assessed Wisdom Scale. Assessment occurred at baseline (T_0_), end of treatment (T_1_), and 4 (T_2_) and 8 months later (T_3_). Overtime effects of NT on wisdom scores were assessed using latent growth curve models with time-invariant covariates for impact.

**Results:**

The intervention (NT) group showed significant, sustainable overtime within-group improvement in perceived wisdom. Furthermore, when compared to the control group, the NT group showed significant immediate improvements in perceived wisdom [*F*(2.726, *p* = .041)], which were maintained at all follow-up points. This effect remained after controlling for age, gender, and educational level (*T*_ML_(11) = 17.306, *p* = .098, root mean square error of approximation = 0.079, comparative fit index = 0.960). No adverse reaction was recorded.

**Discussion and Implications:**

NT underpinned by a ToL methodology offers a new theory to understand, promote, and appreciate perceived wisdom in older Chinese living in HK. It contributes to psychotherapy and professional social work practice for older Chinese.

**Translational Significance:** Older adults generally accumulate valuable wisdoms throughout their lives, but neither they, nor others, may recognize the value of these. This may constrain problem-solving, self-esteem, and relationships with others. In four biweekly sessions, older Chinese adults participated in collaborative conversations with a narrative therapist to reexamine their life experiences and recognize their accumulated wisdom. The intervention significantly improved self-perceptions of wisdom compared to baseline, and a control, with short- and longer-term effects. Narrative therapy could be employed to assist older adults to recognize the value of their wisdoms, to enhance their self-worth, and participation in family and community.

The topic of “wisdom” is a growing field of research in psychology and social gerontology (Glück, 2018; Glück et al., 2013; Staudinger & Glück, 2011). It is a concept that has been documented for many years as a source of human strength (Erikson, 1997). It has attracted increasing empirical investigations in relation to positive psychology (Fung et al., 2020; Luna et al., 2017). Despite pioneering work conducted in the 1970s and early 1980s (Bangen et al., 2013; Ferrari & Weststrate, 2013; Seligman & Csikszentmihalyi, 2000; Staudinger & Glück, 2011), it is only recently that consensus has been reached on the definition of the concept of wisdom (Jeste et al., 2010). It is described as a multidimensional construct learned and developed through reflection of life lessons, which enables individuals not only to grow individually, but also to positively contribute to the common good. In fact, life wisdom has been found to have a profoundly positive influence on older peoples’ life satisfaction, independent of other life circumstances (Ardelt, 2004). Research also suggests that wise individuals age more successfully than people with less wisdom (Ardelt, 2000; Cheung & Chow, 2019).

The development of wisdom in older Chinese is largely related to critical life events. Wisdom in old age is an attitude toward life, developed through reflection, after individuals have experienced both favorable and unfavorable life circumstances (Ardelt & Jeste, 2018; Chow, 2018). In contrast to the leading Western definition of wisdom, the concepts of Chinese wisdom place less emphasis on expert knowledge and intelligence. Instead, accumulation of life experiences is viewed as a dominant determinant of wisdom (Webster, 2003, 2007). Chinese people tend to emphasize reflective aspects, including self-reflection and self-insight (Ardelt, 2003; Webster, 2007). Reflections on life lessons over their life span result in wisdom (Ardelt & Jeste, 2018; Jeste et al., 2010). Consistent in part with the Western model of wisdom, components of Chinese wisdom include aspects of cognitive understanding about life (Ardelt, 2003; Cheung & Chow, 2019).

Concepts of Chinese wisdom distinguish between individual and collective wisdom. The current discussion in research largely focuses on individual wisdom (Ardelt, 2003; Glück & Bluck, 2013; Webster, 2007). Collective wisdom is seldom mentioned or investigated, and thus requires further exploration. Collective aspects of wisdom demonstrate the cultural collective nature of the Chinese population (Cheung & Chow, 2019). Old age itself is not a sufficient condition for the development of wisdom; rather, life experiences, and the readiness of society to accept wisdom associated with old age are more important, and relevant to the discussion (Ardelt & Jeste, 2018; Jeste et al., 2010; Staudinger & Glück, 2011).

In line with theories of lifelong psychosocial growth and life course principles of human development and agency (Schriver, 2020), older adults generally possess rich and valuable wisdoms concerning many important aspects of life. However, how best to preserve and transfer wisdom for the common good remains unanswered (Webster et al., 2014). There is little empirical evidence on whether the wisdoms of older adults can be harnessed for their own benefit as well as for others. This may be due to physiological decline associated with aging, and pervasive societal ageist attitudes (North & Fiske, 2015). The current health and social care training primarily focuses students on people’s problems, symptoms, and deficits, instead of their efficacy, wisdom, and coping strengths. Thus, the use of language and diagnostic deficits may disempower older people by saturating them with pathologies. This is likely to totalize the persons with problem-saturated life events, and adversely affect their sense of self and their aging identity. With proper methods for wisdom rediscovery and recollection, and an appropriate platform to share wisdoms with others, the wealth of wisdoms of older people might be leveraged for greater societal good.

Over the past few decades, an increasing body of literature has consistently shown that wisdom is positively related to individual eudemonic and hedonic well-being. Wisdom scores predict levels of generativity, ego integrity, and positive psychosocial values, such as personal growth and sense of coherence (Webster, 2007, 2010). This is also related to civic engagement and altruism (Bailey & Russell, 2012), benefit-finding in terminally ill patients (Costa & Pakenham, 2012), and forgiveness and psychological well-being (Taylor et al., 2011). Hence, identifying an intervention with empirical evidence of a positive impact on the recognition, rediscovery, and rejuvenation of older adults’ life wisdom will be an important addition to the current scant body of evidence.

## Wisdom and Narrative Therapy

Narrative therapy (NT) emphasizes personal experience and elaboration of meaning, and has attracted increasing attention in the practice of psychotherapy and social work (Danner et al., 2007). NT primarily focuses on people’s personal expression of their life experiences (Freedman, 1996; Madigan, 2019; Morgan, 2000; White, 2007). NT views people as experts in their own lives, who possess abilities, knowledge, and wisdom that assists them to cope with difficult life situations. It emphasizes the importance of an individual’s subjective perception and experiences of a problem, construction, and co-construction of the relative nature of reality and being, as well as enduring problem-solving capacities built on experiences of previous successful coping. Wisdom has been linked to increased self-knowledge in the context of autobiographical reasoning (Randall, 2011), and the appraisal of life lessons for personal growth and common good (Jeste et al., 2010). NT assumes that throughout their lives, people accumulate critical elements of personal inner resources, including knowledge, beliefs, values, hopes, loves, competence, and commitments (White, 2007). Thus, NT offers a persuasive paradigm of practice to identify and recollect those critical elements, apply them to manage individual life challenges across the life span, and to convey them in a manner that will benefit others at societal, familial, and individual levels.

Narrative gerontology (de Medeiros, 2014; Randall & Kenyon, 2004) sheds light on new ways of responding to the stories of multiple identities assumed by older adults throughout their lives. It is essential to delineate the backbone of the narrative with research on reminiscing and life review to distinguish their uniqueness before NT can be further deliberated and applied. While reminiscence is a recall of memories, which is usually a pleasurable experience of the narrator, life review is a critical analysis of one’s life history. By examining each developmental stage, it helps to resolve past conflicts and overcome unsuccessful earlier life events in achieving ego integrity (Josselson, 2006). Through collaborative conversations, narrative therapists engage people to freely recollect important incidents in life in the process of reauthoring their life stories to accommodate transitions and challenges. This preserves their preferred identity and reconstructs their future (White, 2007).

This study applied an innovative NT-based intervention embedded in an inclusive theoretical framework to help older Chinese living in Hong Kong (HK) to recognize, better understand, and better reconnect with their wisdom, and to provide opportunities for them to transfer their wisdom for the benefit of others in HK. The paper reports on the intervention itself, and its short- and longer-term effectiveness.

## Hypothesis

The group that received NT fused with Tree of Life (ToL) methodology would demonstrate a significantly greater positive change on wisdom scores, compared with the control group.

## Method

### Study Design

Single-blinded randomized controlled trial (RCT).

### Reporting Criteria

**Figure 1.**
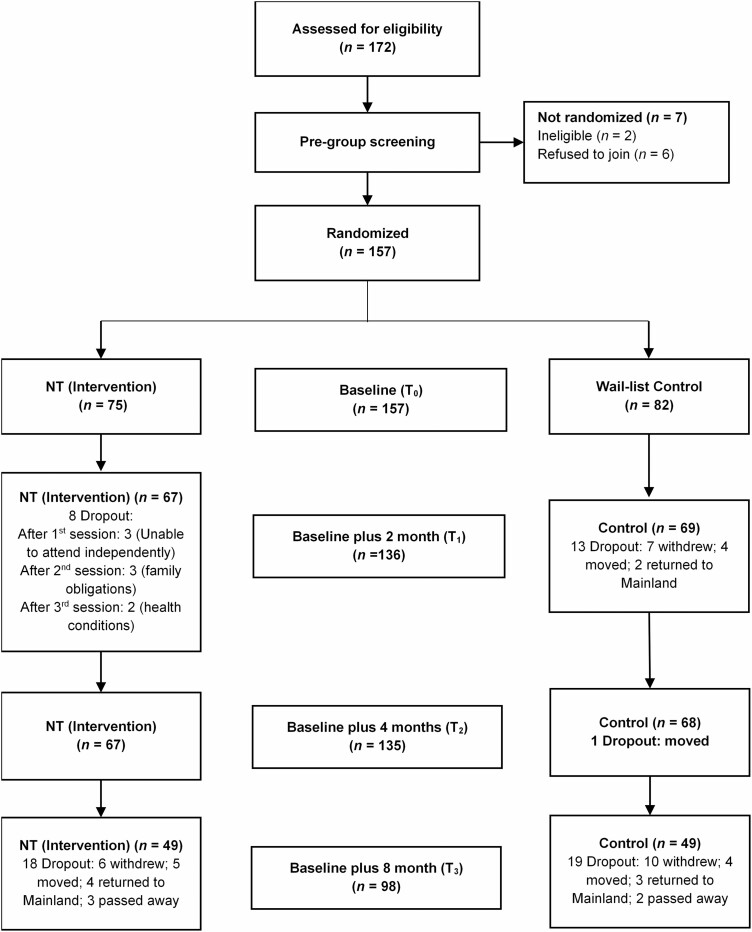
CONSORT table of the study. NT = narrative therapy.

This study was reported in line with the CONSORT statement (see Figure 1).

### Theoretical Framework

The intervention used the ToL methodology, which is based on collective narrative practice. The metaphor of ToL has been widely used clinically, initially with children (Ncube, 2006), and subsequently extended to young and older adults (Denborough, 2004, 2008; Dickson, 2009). It was initially developed as a response to trauma, but it is also a useful tool for wider conversations for nontherapeutic purposes, such as intergenerational acknowledgment and environmental education (Denborough, 2008). This is the first attempt to use the ToL metaphor as a method to illuminate, rediscover, and recollect the life wisdom in the context of HK. The use of ToL is a “good fit” with the narrative approach to understanding wisdom accumulation over one’s life span. By examining one’s past and present, the many aspects of one’s life and self-identity are symbolized, reflected, and embedded in the different parts of the tree. It addresses issues of losses, strengths, and ups and downs in one’s life (Chow & Nelson-Becker, 2010). Exploring wisdom via ToL is also made possible by identifying older adults’ future hopes and dreams and identifying ways in which they plan to achieve them based on the wisdoms that they have learned from the past. The metaphor of ToL is a sound, integrative, and respectful approach to enable older adults to speak about different aspects of their lives.

### The ToL Conversations

The ToL process consists of four sessions, each of which lasts for 2 hr (see online Supplementary Material for the protocol I and II):

Session 1: Tree of Life;
Session 2: Forest of Life;
Session 3: When the Storms Come; and,
Session 4: Celebration of Life.

### Participants and Procedures

The target population was Chinese people aged 55 and older, living in different major HK regions: Kowloon, New Territories, and HK Island. Inclusion criteria were that they: (a) had experienced at least two critical life experiences/life lessons; (b) were able to perform basic daily functional activities independently; (c) were physically mobile (including with walking aids or a wheelchair); (d) possessed normal cognitive ability; (e) did not experience active psychotic symptoms, such as hallucinations or delusions; (f) were not currently experiencing acute crisis with severe stress; and (g) were willing to work in small groups.

### Sample Size Calculation

The sample size was determined by power analysis (Cohen, 1992). Ryan et al. (2006) found that the estimated effect size of NT intervention is 0.4. The standardized difference (0.4) was calculated assuming a standard deviation (*SD*) of 5, which was estimated using references from those working with older adults (Shyu et al., 2008). With a 5% significance level (two-tailed) and power of 80%, a sample size of 72 is estimated. Since a control group of similar size was needed, the recruited sample size was set as 144. If there are six participants in a group, at least 12 intervention groups and 12 waitlist control groups are required during the study period.

Institutional Review Board approval was obtained from the City University of Hong Kong. Informed written consent was sought from all participants before commencing the trial, and consent was confirmed at each intervention session.

Invitations were sent to 41 District Elderly Community Centers (DECCs) to recruit potential participants. Nominees were invited to the City University of HK for a prestudy group interview to confirm eligibility for study enrollment. Exclusion criteria were that potential participants: (a) tested poorly for cognitive capacity, using the Mini-Mental State Examination with a cutoff score at 23 (Blake et al., 2002); (b) manifested active psychotic symptoms, such as hallucinations or delusions (which could pose threats to other members or divert group processes); (c) were intellectually challenged or suffered from personality disorders (which might render them unable to grasp what was being imparted, or may be too critical or rigid to participate fully in group processes); (d) lacked motivation to work on problems (as motivation was crucial for participants to ensure that they attended all group sessions); and (e) had a record of one or more suicidal attempts or violent behavior (which may negatively affect the small group environment of the intervention).

Eligible consenting participants were randomly assigned to pairs using a computerized method of minimization (Pocock, 1983). One of each pair was then randomly assigned to the intervention group and the other to the waitlist control group. To minimize uneven distribution of known variables, randomization occurred after stratification by age group (55–65 years, 66–75 years, 76–85 years), as well as region of residence.

### Intervention

The intervention was delivered in four biweekly sessions conducted at 12 centers located around HK. Participants allocated to the intervention group were divided into 12 small groups that met at one of the centers (usually the closest to their home). The waitlist control participants received no intervention, but they were invited to join the intervention when the study was completed. Two narrative therapists with advanced NT training and extensive experience were recruited to lead the NT groups, with each session having specific concepts, objectives, and implementation plans with defined details (see the protocol in the online Supplementary Material for detailed practice).

The intervention was assessed for the fidelity of implementation (FOI) through pregroup briefing, training, and videotaping all group sessions, coupled with biweekly peer debriefing and supervision provided by the PI, to ensure the procedure was standardized (Gearing et al., 2011). To evaluate the FOI, we adopted the method proposed by Keith et al. (2010) to compare the differences in outcomes observed among the centers and between therapists.

Participants were offered a coupon worth HK$40 (i.e., US$5) after each session, as compensation for transport, travel costs, and inconvenience, or recompense for extraordinary household arrangements (such as requiring care for an ill or older relative, or grandchildren). Participants who completed four sessions received HK$160 (US$20) at the final session to cover additional costs.

### Waitlist Control Group

We adopted a waitlist control design as we wanted to ensure that all eligible participants could receive the intervention. The waitlist control group was offered access to NT after the study finished.

### Study Measurement Procedure

Trained (blinded) interviewers conducted pretest/baseline interviews and quantitative assessments (T_0_) for all participants. After the intervention had been delivered, all participants were again assessed at a postintervention interview at 2 months (T_1_), at 4 months (T_2_), and 8 months (T_3_) postintervention. Narrative conversations of each session throughout the intervention and assessment phases were documented for clinical evidence, including attentiveness, involvement, and responsiveness.

### Measures

A purpose-built questionnaire sought sociodemographic information from participants, including age, gender, location of residence, and educational level. Perceived Wisdom was measured items from the Self-Assessed Wisdom Scale (SAWS; Webster, 2003, 2007). SAWS is a 40-item questionnaire reflecting the five components of wisdom (critical life experiences, reminiscence/reflectiveness, openness to experience, emotional regulation, and humor). Participants responded to each question using a Likert-type scale, where 1 reflected “strongly disagree” to 6 reflecting “strongly agree.” The SAWS has been shown to have good reliability (i.e., test–retest and internal consistency) and different forms of validity (Thomas et al., 2019). In response to recent controversies related to the factorial validity of SAWS (Alves et al., 2014; Aredelt, 2011a; Bangen et al., 2013; Taylor et al., 2011; Webster, 2003, 2007; Webster et al., 2011), this study used the nine-item Brief SAWS (BSAWS; Fung et al., 2020). This comprised SAWS items 6, 18, 22, 23, 27, 29, 34, 36, and 40. The BSAWS has been shown to adequately capture the five SAWS domains. For this study, the BSAWS was back-translated into Chinese (Brislin, 1970; Cha et al., 2007) and the internal reliability was confirmed by Cronbach’s α at 0.81 (Cronbach, 1951) and McDonald’s omega at 0.81 (McDonald, 1999; Revelle & Zinbarg, 2009; Zinbarg et al., 2005).

### Data Analyses

Descriptive statistics were generated for the demographic and clinical variables. Assuming group equivalence at baseline and using an intention-to-treat approach (Alshurafa et al., 2012; Hollis & Campbell, 1999; Lachin, 2000; Montedori et al., 2011), independent within-group *t* tests were performed to test the immediate effect and the long-term (sustainability) effect of the treatment, at each measurement time frame (2, 4, and 8 months posttreatment; i.e., T_1_, T_2_, and T_3_). Repeated-measures analyses of variance (ANOVAs) with a Greenhouse–Geisser correction (Girden, 1992; Greenhouse & Geisser, 1959) determined differences between intervention and control groups at the different time points. Finally, structural equation modeling latent growth curve models with maximum likelihood estimation were used to evaluate the impact of NT on respondents, considering repeated measurements on perceived wisdoms and other contextual variables (Cheong et al., 2003; Duncan & Duncan, 2004; Jones, 2012; Long, 2012; McArdle & Epstein, 1987).

Model 1 was an unconditional latent growth curve model fitted to analyze the effect of overall intervention on perceived wisdom in different time frames (T_0_–T_3_). Latent growth curve modeling with time-invariant covariates for impact of NT on wisdom scores (Models 2, 3, and 4) were performed to examine if the trajectories varied by conditions, including repeated measurements nested within participants, and participants within experimental conditions, age, gender, and educational attainment. As suggested in the structural equation modeling literature, the chi-square value may be subject to the effect of sample size (Bentler & Bonett, 1980; Kline, 2005); hence, the following fit indices were used to determine whether the model had an adequate fit, that is: comparative fit index (CFI) > 0.950, standardized root mean square residual (SRMR) < 0.080, and root mean square error of approximation (RMSEA) < 0.080 (Browne & Cudeck, 1993; Hu & Bentler, 1999). The cases with missing data were handled with the maximum likelihood procedure (Collins et al., 2001; Schafer & Graham 2002).

All analyses were computed with IBM SPSS 26.0 and R computing environment version 3.6.1 with lavaan package 0.6-5 (Rosseel, 2012).

## Results

### Fidelity of Intervention

None of the outcome measures differed significantly between centers or therapists, indicating that the sessions were run similarly despite location and leader.

### Sample

Over 240 participants made inquiries about study participation or were nominated by one of the 18 participating DECCs. One hundred seventy-two potentially eligible participants agreed to be screened for eligibility, and of these, 157 were deemed to be eligible and consented to join the study (91.28% recruitment). Table 1 reports participant demographic characteristics and Figure 1 reports the CONSORT diagram.

**Table 1.** Participant Demographic Characteristics

Variables	NT group (*n* = 82)	Control group (*n* = 75)	Overall (*n* = 157)
Age, mean (*SD*)	73.12 (9.12)	71.99 (7.90)	72.6 (8.55)
Gender, *n* (%)			
Male	21 (25.6%)	19 (25.3%)	40 (25.5%)
Female	61 (74.4%)	59 (74.7%)	117 (74.5%)
Education level, *n* (%)			
No formal education	14 (17.1%)	12 (16.0%)	26 (16.6%)
Primary education	23 (28.0%)	27 (36.0%)	50 (31.8%)
Secondary education	28 (34.1%)	17 (22.7%)	45 (28.7%)
Tertiary education	16 (19.5%)	14 (18.7%)	30 (19.1%)
Missing	1 (1.2%)	5 (6.7%)	6 (3.8%)
Marital status, *n* (%)			
Single	9 (11.0%)	6 (8.0%)	15 (9.6%)
Married	35 (42.7%)	29 (38.7%)	64 (40.8%)
Divorce/separated	8 (9.8%)	7 (9.3%)	15 (9.6%)
Widowed	29 (35.4%)	33 (44.0%)	62 (39.5%)
Other	1 (1.2%)	0 (0.0%)	1 (0.6%)

*Notes*: NT = narrative therapy; *SD* = standard deviation.

Among the *N* = 157 participants who commenced the study, 21 (13.38%) dropped out before the posttreatment (T_1_) data collection. This was within acceptable guidelines, particularly given the age of participants (Schulz et al., 2010). Among the 75 intervention group participants, eight (*n* = 8; 10.67%) dropped out at T_1_ (posttreatment, 2 months after baseline): three (37.5%) dropped out after the first session as they were unable to travel to the intervention center by themselves; three (37.5%) dropped out after the second session because of family obligations; and two (25%) dropped out after the third session because of health concerns: one was hospitalized, and another had to rest at home. Thus, there was 89.33% successful participation in all four sessions.

There was a higher dropout rate among 82 waitlist control group participants: 13 (*n* = 13; 15.85%) from the waitlist group at T_1_ (2 months after baseline): seven (53.85%) decided to withdraw because of other commitments; four (30.77%) moved; and two (15.39%) returned to Mainland. The dropout rate was stable during T_2_ data collection (4 months after baseline): no dropout for the intervention group, and only one from the waitlist control group because the participant was moved. By the last measurement point (T_3_, i.e., 8 months from baseline), *n* = 98 were measured (five [3.7%] had died, seven [5.6%] had returned to Mainland China, and nine [6.6%] had moved residence) and were unable to be traced.

There was no significant between-group difference in any demographic. Many participants were female (intervention group 74.4%, control group 74.7%). The mean age in the intervention and control groups was 73.12 years (*SD* = 9.12) and 71.99 years (*SD* = 7.90), respectively.

Table 2 reports the outcome measures for both groups over the study period. Overall, mean scores for the perceived wisdom measure differed significantly between time points between the intervention and control groups, *F*(2.726, 256.228) = 2.894, *p* = .041. There was no significant between-group difference in wisdom scores at T_0_. The intervention group showed significant within-group improvement in perceived wisdom immediately after the intervention (T_1_) and maintained this improvement over time (at 4 months [T_2_] and 8 months [T_3_]). There was no difference between groups at T_1_; however, significant within-group differences of all outcome variables were observed at both 4 months (T_2_) and 8 months (T_3_) follow-up, with the intervention group showing greater improvement.

**Table 2.** Mean Differences Between Intervention and Control Group by Outcome Measures

Time intervals	Wisdom scores		
	Control group (*SD*)	Experimental group (*SD*)	All (*SD*)
Baseline (T_0_)	35.17 (9.66) (*n* = 75)	35.85 (8.69) (*n* = 82)	35.53 (9.13) (*n* = 157)
Baseline plus 2 months (T_1_)	35.69 (8.97) (*n* = 67)	37.51 (7.86) (*n* = 69)	36.61 (8.45) (*n* = 136)
Baseline plus 4 months (T_2_)	35.90 (8.67) (*n* = 67)	39.49 (6.08) (*n* = 68)	37.70 (7.66) (*n* = 135)
Baseline plus 8 months (T_3_)	36.50 (10.47) (*n* = 49)	39.06 (7.22) (*n* = 49)	37.78 (9.04) (*n* = 98)

*Note*: *SD* = standard deviation.

The hypothesis was thus supported, as those who received NT fused with ToL methodology demonstrated a significant positive change in wisdom scores compared with the waitlist controls.

Table 3 reports the results of structural equation modeling of latent growth curve models. Model 1 supports the significant impact of NT on wisdom scores (from ANOVA models). To further examine the effectiveness of NT on wisdom scores, Models 2 and 3 attempted to include conditions such as control versus experimental (without specifying the time points) and demographic variables, such as age, gender, and educational level. None of these factors significantly affected on the wisdom scores.

**Table 3.** Results for Unconditional and Conditional Latent Growth Curve Models for Impact of Narrative Therapy on Wisdom Scores

Variables	Model 1	Model 2	Model 3	Model 4
Means or intercepts				
*i* (intercept)	36.119*	35.502*	43.887*	43.811*
*s* (slope)	0.760*	0.432	−1.979	−1.949
Variance residual variance, and covariance				
*i* (intercept)	42.683*	42.274	39.606*	39.595*
*s* (slope)	0.895	0.746	1.135	1.197
Covariance of *s* and *i*	−0.844	−0.982	−1.728	−1.736
Covariate regressions				
*i* on				
Age at baseline			−0.183	−0.182
Gender (female vs male)			0.683	0.683
Education level			1.462	1.458
Group (control vs experimental)		1.194	1.355	
*s* on				
Age at baseline			0.033	0.033
Gender (female vs male)			−0.126	−0.127
Education level			0.095	0.094
Group (control vs experimental)		0.644	0.602	
T_0_ BSAW * group (control vs experimental)				1.185
T_1_ BSAW * group (control vs experimental)				1.446
T_2_ BSAW * group (control vs experimental)				3.250*
T_3_ BSAW * group (control vs experimental)				2.366
Model fit statistics				
Model chi-square	9.819	11.223	20.063	17.306
Degree of freedom	5	7	13	11
CFI	0.969	0.973	0.956	0.960
RMSEA [90% CI]	0.101 [0.000–0.194]	0.080 [0.000–0.162]	0.077 [0.000–0.140]	0.079 [0.000–0.147]
SRMR	0.049	0.044	0.047	0.045
*N*	95	95	92	92

*Notes*: BSAW = Brief Self-Assessed Wisdom; CFI = comparative fit index; CI = confidence interval; RMSEA = root mean square error of approximation; SRMR = standardized root mean square residual. All covariates are mean-centered.

*p < .05.

Time-invariant covariates, that is, T_0_, T_1_, T_2_, and T_3_, were introduced in the conditional latent growth curve model (Model 4). There were no differences between experimental and control groups’ wisdom scores (*SE*) = 1.185 (*p* = .504) after the first intervention (T_1_). Notably, the control group demonstrated significant wisdom score differences with the intervention group at T_2_, with *SE* = 3.250 (*p* = .027). This effect was controlled with age (*SE* = 0.033, *p* = .873), gender (*SE* = −0.127, *p* = .389), and education level (*SE* = 0.094, *p* = .792). The model also indicated good fit, with (*T*_ML_(11) = 17.306, *p* = .098, RMSEA = 0.079, CFI = 0.960, SRMR = 0.045). All the above models did not rely on correlating the error terms to fulfill the requirements of acceptable fit. There were no adverse reactions from study participation.

## Discussion

This study is the first to apply an RCT design to evaluate the effectiveness of a theoretically developed NT intervention on recollecting and improving perceived wisdom in older Chinese living in HK. Compared with being on a waiting list, an NT intervention infused with ToL metaphor was effective in supporting older adults to recognize, better understand, and better reconnect with their wisdom, in the short and longer term. The findings thus concur with current evidence, which suggests that wisdom can be enhanced by reflecting on critical life experiences; wisdom scores are positively associated with well-being, mastery, hope, and meaning in life; and the benefits of an intervention to enhance wisdom can be preserved over time (Ardelt, 2011b; Fung et al., 2020; Glück & Bluck, 2013; Webster, 2007; Webster & Deng, 2015).

The underpinning tenet of NT views people as wise and resourceful, and posits that wisdom is accumulated through life lessons and experiences, consistent with theories of psychosocial and life-span development. ToL methodology emphasizes the use of strength- and meaning-based construction to rescue and record older people’s problem-solving capacities, principles and purpose in life, and successful coping. For example, in identifying the “aerial roots” from older trees, older people may find “prop roots” that mature into thick, woody trunks, which with age, can become indistinguishable from the primary trunk. Our study provides support for the safe use of NT for Chinese older adults in HK to positively improve their perceived wisdom, like prop roots, which uphold and become beneficial to their general physical and mental health, and validate their wisdom of life (Bailey & Russell, 2012; Costa & Pakenham, 2012; Webster, 2007, 2010).

Another contribution of this study to the body of knowledge is providing empirical evidence to support the effectiveness of an NT intervention fused with ToL methodology to rediscover life wisdom. The effects of NT in the existing literature have mainly been measured by qualitative outcomes (Denborough, 2008; Dickson, 2009; Hardtke & Angus, 2004; Josselson, 2006; Kogan & Gale, 1997; White, 2007). This study attempted to quantitatively measure perceived wisdoms in older adults, which paves the way to study the effect of NT on other populations in different societies (Semmler & Williams, 2000).

There are significant clinical implications from this study, particularly in recollecting enhanced wisdom. These include: (a) rediscovering older adults’ wisdom formally and systemically; (b) witnessing “wisdom” in practice to enhance and renew older adults’ self-worth and preferred identity, thus promoting “aging with dignity”; (c) encouraging the practice of “wisdom recognition” to enhance participation of older adults in groups and community, thus promoting “active aging”; (d) promoting the practice of “wisdom transfer,” which encourages peer and cross-generational sharing of effective life management and life planning to promote “productive aging”; (e) enhancing public awareness and recognition of the importance of older adults’ wisdom as critical “social capital”; (f) acknowledging the potential contributions of older adults to society and human development as “positive aging”; (g) recognizing the importance of “wisdom transfer” to revitalize respect and appreciation for older adults at both societal and familial levels, and promoting “respectful aging”; and (h) acknowledging that the importance of “wisdom transfer” is likely to facilitate the development of innovative holistic public aging policy initiatives.

The ToL metaphor provided a vivid and coherent framework in narrating different aspects of oneself, and one’s life externally, with ample space to understand, accommodate, and transcend the differences, and organize these human experiences in the context of individual worldviews within the framework of existential philosophy. Gaining the understanding of therapists’ own worldview, and participants’ (who come to consult the therapist) worldview are key elements in enhancing cross-cultural effectiveness. Different ToL metaphors can be found in different parts of the world. Adopting the ToL metaphor, which initially evolved in Africa (Ncube, 2006), is an advanced method by which to rediscover life wisdom for Chinese older adults (Chow & Fung, 2020). This highlights the ToL’s capacity to innovate and broaden thinking in cross-cultural practice, as well as further strategies to enrich cross-cultural counseling and psychotherapy.

Strengths of the study are the high level of interest in participating in it, from older Chinese, its 91.28% response rate during recruitment, and the high retention rate throughout the study. Those who dropped out all had valid reasons for doing so (health, family obligations) rather than disinterest in the therapy. Moreover, the attrition rate from the intervention group was similar to the recently reported longitudinal RCT of older adults in HK (Jiang et al., 2019).

However, this study has limitations. There are more females than males utilizing DECC services; therefore, the sample recruited for this study potentially represented those women who were more active and sociable, and who wanted to participate in community services. Also, as the study had an exclusion criterion of lack of motivation, this may have limited the external validity of study findings. Finally, there was a higher dropout rate (15.85%) from the waitlist control groups at T_1_ (post-test, 2 months after baseline) compared to the intervention group (10.66%). We hypothesize that those in the waitlist did not want to wait until the intervention group had completed all assessments. We recommend that the next research investigating NT in this population adopts a more active control group, such as a treatment as usual practice, so that participants in all groups experience concurrent parallel interventions.

## Conclusion

This RCT provides support for NT, coupled with a new paradigm of clinical practice (ToL metaphor) on significantly, safely, and sustainably improving and consolidating wisdom in older Chinese living in HK, compared with a waitlist control group. The intervention effects lasted up to 8 months postintervention. These findings have profound theoretical implications for professional psychotherapy and social work practice, in terms of a strength- and meaning-based approach to late-life development, as well as proposing a new theory in understanding wisdom in older adulthood.

## Supplementary Material

igab027_suppl_Supplementary_Materials
