# Supplementary material for: Narrative Group Intervention to Rediscover Life Wisdom Among Hong Kong Chinese Older Adults: A Single-Blind Randomized Waitlist-Controlled Trial
Source: Innov Aging. 2021 Aug 2;5(3):igab027. doi: 10.1093/geroni/igab027 (PMC8489428; doi:10.1093/geroni/igab027)
Supplement: igab027_suppl_Supplementary_Materials [file igab027_suppl_supplementary_materials.docx]

**Tree of Life Protocol I: Detailed session plan of Tree of Life** (Carey & Russell, 2003; Choi & Landeros, 2011; Edmondson, 2005)

| **Session 1: Tree of Life**   - The aim of this session is to allow each older adult to establish “a second story” about his or her life. - The session begins with a brief discussion about trees in general. The ToL metaphor is presented and explained to participants, who will draw (with assistance from a helper, if necessary) their own ToL in accordance with the following parts of a tree: (1) roots; (2) ground; (3) trunk; (4) branches; (5) leaves; (6) flowers; (7) fruits; (8) aerial/ prop roots, and (9) winged seeds (see Protocol II: different part of a tree). |
| --- |
| **Session 2: Forest of Life**   - The aim of this session is to share different stories about trees and the contextual factors of the forest in the group. - After participants have completed their ToL drawing, a “forest of life” is created, and participants will be invited to share their stories about trees. The investigators may ask questions associated with each part of a tree and the contextual factors which may affect the forest (see Protocol II). |
| **Session 3: Storms of Life**   - The aim of this session is to create space for participants to speak about some of the difficulties or hardships that they have experienced and how they made it through. - This session begins by drawing older adults’ attention to the hazards and their effects that trees and forests sometimes confront in life, such as fire and storms. The particular focus of the session is to identify ways utilized by participants to respond to challenges in their lives. In this regard, White’s (2007) re-author conversations that highlight “unique outcomes” are used to explore elements of participants’ responses to these challenges in more details. - The following line of questioning is employed: (1) identifying difficulties or hardships (“known and familiar”); (2) negotiating an experience and particular definition of the unique outcome/solution; (3) mapping the effects and potential effects of the unique outcome/solution; (4) evaluating the effects of the unique outcome/solution; and (5) justifying the evaluation. |
| **Session 4: Celebration of Life**   - This session is a certificate-giving ceremony. Each participant is given a ToL Certificate to document his or her knowledge, strengths, skills, talents, hopes and dreams, and the like. Before the ceremony, participants are prompted to speak about their future hopes and dreams and their plans to ensure that these will be realized. - Family members and friends are invited as the audience/witnesses, and given the opportunity to listen to, acknowledge, and receive the “life wisdom” as manifested. They will listen to participants’ conversations about their life narratives, and collectively witness and document their strengths. In this way, valuable learning known as their life wisdom is realized and transferred to each participant’s family and friends, and other participants’ family and friends. - The audience/witness members are invited to retell what they have heard to participants and acknowledge the usefulness of their responses to hardships in life. Through the process of tellings, and retellings, it is expected that both the participants and audience/witnesses will recollect, and gain recognitions of their “wisdom” learned from the past, to celebrate with the participants as they acknowledge the benefit of this wisdom. |

**Protocol 2:** Different parts of a tree (Ncube, 2006; Denborough, 2008; Ames, 2008)

| **Part of tree** | **Descriptions** |
| --- | --- |
| Roots | Where participants come from (e.g., country of origin, place of birth); their family history; family members; what they did/do for a living; their careers, just name a few. |
| Ground | Where participants currently live; What are their daily activities. The following can be discussed (appropriately):  - Why do you do this every day?  - What has sustained you doing this regularly? |
| Trunk | Participants’ strengths, skills, talents, personality characteristics, and problem-solving mechanisms.  The following can be discussed, where appropriate:  - How did you develop these strengths, skills…?  - How important are these strengths, skills… in your life? |
| Branches | Hopes, dreams, and wishes that participants have for his or her life. The following can be discussed, appropriately:  - Why are these hopes, dreams, and/or wishes important to you?  - What has sustained your hopes, dreams, and/or wishes?  - Is there a history behind these hopes, dreams, and/or wishes? |
| Leaves | People who are or were important in the participant’s life. The following can be discussed, where appropriate:  - Why is or was this person important to you?  - What was special about this person to you? |
| Flowers | Participants’ cherished memories of beauty, love, connections, and fulfilment. The following can be discussed, appropriately:  - Why these are cherished memories to you?  - What is special about these memories to you? |
| Fruits | Gifts that the participants have been given, may be material gifts or acts of love and caring from others. The following can be discussed, appropriately:  - Why do you think the person gave you this?  - What did they appreciate about you that would have led them to do this?  - What do you think you might have contributed to their life? |
| Aerial/ Prop roots | Strengths that the participants have identified and lived by through earlier life challenges. These may be values, beliefs, commitments in life that they have recognized or acknowledged and testified, where appropriate:   - What uphold you during the trying times? - Tell us a story when you first realize them? Have been supporting you subsequently? |
| Winged Seeds | Participants’ legacies. The following can be discussed, appropriately:  - Why do you think these legacies are important?  - What care and support do you think that you need to ensure that these legacies are carried forward? |
